# Genetic variability of *Mycobacterium tuberculosis* complex in patients with no known risk factors for MDR-TB in the North-eastern part of Lima, Peru

**DOI:** 10.1186/1471-2334-13-397

**Published:** 2013-08-28

**Authors:** Francesca Barletta, Larissa Otero, Jimena Collantes, Belisa Asto, Bouke C de Jong, Carlos Seas, Leen Rigouts

**Affiliations:** 1 Instituto de Medicina Tropical Alexander von Humboldt, Molecular Epidemiology Unit - TB, Universidad Peruana Cayetano Heredia, Lima, Peru; 2 Prince Leopold Institute of Tropical Medicine, Antwerp, Belgium; 3 University of Antwerp, Antwerp, Belgium

**Keywords:** *Mycobaterium tuberculosis*, Haarlem, Genotypification

## Abstract

**Background:**

The aim of this study was to investigate the genetic diversity among *Mycobacterium tuberculosis* complex circulating in patients with no known risk factors for multi-drug resistant (MDR) tuberculosis (TB) living in a high MDR burden area and analyze the relationship between genotypes, primary drug resistance and age.

**Methods:**

Samples were collected during January-July 2009. Isolates were tested for drug susceptibility to first-line drugs and were genotyped by spoligotyping and the 15-loci Mycobacterial Interspersed Repetitive Unit (MIRU15).

**Results:**

Among the 199 isolates analyzed, 169 (84.9%) were identified in the SpolDB4.0 and 30 (15.1%) could not be matched to any lineage. The most prevalent lineage was Haarlem (29.6%), followed by T (15.6%), Beijing (14.1%), Latin American Mediterranean (12.6%) and U (8.5%). A few isolates belonged to the X and S clades (4.5%). Spoligotype analysis identified clustering among 148 of 169 isolates, whereas with MIRU15 all isolates were unique. Out of 197 strains; 31.5% were resistant to at least one drug, 7.5% were MDR and 22.3% showed any resistance to isoniazid.

**Conclusion:**

In contrast with other Latin-American countries where LAM lineage is the most predominant, we found the spoligotype 50 from the Haarlem lineage as the most common. None of the prevailing lineages showed a significant association with age or resistance to isoniazid and/or rifampicin.

## Background

Tuberculosis (TB) currently holds the seventh place in the global ranking of causes of death [1]. In 2010, there were 8.8 million incident cases of TB or more than 24 000 per day [2]. Although Peru accounts for only 3% of the population of the Americas 12% of the region’s TB patients reside in Peru, and 32% of the multi drug resistant (MDR) TB patients [3]. According to the Peruvian National Tuberculosis Program, 17 264 new cases of pulmonary TB were notified in Peru in 2010 [4]. Most (58%) cases are notified in the capital; in particular in the semi-urban districts in northern Lima, which represent 86% of the reported cases in the capital [5]. San Juan de Lurigancho is the largest and most densely populated district in Lima, with more than one million of inhabitants, a TB incidence of 213/100,000 inhabitants [6] and a primary MDR prevalence of 7% among all TB cases [7]. Otero *et al*. found a high rate of primary MDR-TB (6.3%) [8] in a population with no identifiable risk factors for MDR-TB yet living in this high burden area. It is not clear to what extent the emergence of resistant TB in this low-risk population is due to the clonal spread of a limited number of isolates or rather following a random distribution. Also, predominance of specific genotypes amongst younger inhabitants might indicate recent introduction of this genotype in the population [9]. Molecular epidemiological methods could help us to better understand TB population dynamics in Northern Lima, where antibiotic resistance patterns have increased during recent years [10,11]. However, data on the molecular epidemiology of TB in this country is limited; the SpolDB4.0 spoligotyping database comprises only 96 Peruvian strains of the 39 609 published entries [12].

The aim of this study was to identify predominant circulating TB lineages in this population with no known risk factors for MDR-TB living in a hyper endemic neighbourhood of Lima, and to investigate the relationship between the genotypes, primary drug resistance patterns and age.

## Methods

### Study setting and population

This study was embedded in a prospective cohort of new cases of sputum smear-positive pulmonary TB, conducted in a North Eastern district in the Lima Province in Peru [12]. The district has 33 health centers and one general hospital managed by the Ministry of Health. All these health facilities provide TB treatment and have laboratory facilities to screen TB suspects, such as smear microscopy. For this study, sputum samples were collected from sputum smear-positive pulmonary TB subjects, previously untreated, >18 years, and with no known risk factors for MDR-TB at 34 health facilities [13], including the hospital of the district, between January and July 2009.

Alcohol and drug abuse were considered high risk factors for MDR TB, in addition to the risk factors considered in the national guidelines: persons reporting exposure to a MDR-TB case or to a TB case that failed treatment or that died during treatment; patients with immunosuppressive co-morbidities such as HIV and diabetes; persons working or admitted in a prison; health care workers, and persons with a recent and prolonged admission to a hospital. Patients with at least one of these factors were excluded. All TB patients were treated according to the National TB Guidelines [14] in line with WHO treatment guidelines [15].

The district is geographically located on the slope of a hill and it is informally divided in an upper- and lower area. The upper area is poorer, with light construction houses that typically lack basic necessities (light, water and/or sanitation). The lower area is more urbanised; houses are larger and made of cement.

### Culture and drug-susceptibility testing

Sputum samples were transported to the Microbiology Laboratory at the Institute of Tropical Medicine (IMTAvH, Lima, Peru) within 6 hours of being produced. Samples were cultured on the same day or were kept at 4°C when they arrived in the afternoon or during the weekend. Smear microscopy following the Ziehl Neelsen method was done, and then samples were cultured on two slopes of Löwenstein-Jensen (LJ) medium, following decontamination using the NalC-NaOH method. The *Mycobacterium tuberculosis* (MTB) isolates were tested for drug susceptibility (DST) using the 7H10 agar method with the following drug concentrations: 0.2 μg/ml and 1 μg/ml isoniazid (H), 1 μg/ml rifampicin (R), 2 μg/ml streptomycin (S), and 6 μg/ml ethambutol (E).

### Data collection methods

Data were collected at the health care facilities. Researchers did not interfere with routine patient management. The trained and experienced field workers interviewed the patient to obtain socio-demographic data (name, sex, age), and determine MDR-TB risk factors to exclude patients from this study.

### DNA isolation

To obtain genomic DNA for spoligotyping and MIRU-VNTR typing, mycobacterial colonies grown on LJ medium were resuspended in 100uL 1X Tris-EDTA buffer (10 mM Tris-HCl, 1 mM Ethylenediaminetetracetic acid disodium [pH8.0]) and then boiled for 30 minutes. The suspension was centrifuged at 14 000 rpm for 10-15 minutes to pellet cell debris. The supernatant containing DNA was stored at -20°C and used in PCR reactions.

### Spoligotyping

Spoligotyping was performed using primers (DRa and DRb) corresponding to the direct repeat (DR) region of the genome of *Mycobacterium tuberculosis* according to the procedure described by Kamerbeek *et al*. Amplification and hybridization were performed using an in house prepared membrane [16]. The hybridized membrane was exposed to X-ray film for detection of hybridization signal. The X-ray film (Hyperfilm™ ECL, Amersham Bioscience UK Ltd.) was read manually to obtain a complete pattern of the presence or absence of the spacers between the Direct Repeats harboured by a particular strain. To determine the spoligotype family, patterns were compared to those in the international database of Spoligo patterns (SpolDB4).

### MIRU-VNTR

MIRU-VNTR is a PCR-based typing method that assigns the number of tandem repeats for independent loci (MIRUs) that were found to be polymorphic in MTB. Standardized MIRU-VNTR typing based on 15 loci was performed using the manual method. Each locus was amplified separately by simplex PCR with Qiagen Hotstart Taq Polymerase kit including Q solution, according to the Genotyping of MTB Technical Guide [17]. Products were analyzed by electrophoresis using 2% agarose (Promega, Fitchburg, USA) gels. The H37Rv reference strain was included in each batch of PCRs and gels.

### DNA fingerprint analysis

Demographic information (sex and age), spoligotyping and MIRU-VNTR patterns were compared to the international SpolDB4.0 database using MIRU-VNTR*plus*, freely available web-based software [18]. The patterns obtained received a spoligo-international type (SIT) according to the cluster assignment. MIRU-VNTR profiles with double alleles at a single locus were considered to be clonal variants of the same strain, whereas those with double alleles at 2 or more loci were considered to be mixed infections or the result of cross-contamination [19,20]. Identical spoligotypes and MIRU-VNTR patterns were considered to be in a cluster.

### Data analysis

Patient data, smear, culture and DST results were entered in a dedicated Access database. Quality control was done for 20% of the fields; 100% of those with more than 5% of mistakes and for all DST and molecular (Spoligotyping and MIRU15 analysis) quality control was done for the double entry of the data to the database.

Spoligotype patterns in a binary format were entered in an Excel sheet, and compared with the spoligotype database SpolDB4 using MIRU-VNTR plus. The Hunter Gaston Discriminatory Index (HGDI) was used to calculate the discriminatory power of spoligotyping method [21]. The Chi square or Exact Fisher test when necessary were employed to evaluated differences in age and drug resistance patterns among the lineages. (Epi Info v7; Georgia, USA). Values of p of less than 0.05 were considered significant.

### Ethical consideration

The study was approved at the Institutional Review Board at Universidad Peruana Cayetano Heredia. All enrolled patients signed informed consent. Data was managed anonymously. Smear, culture and DST results were given to the doctor at the health service as soon as they were available.

## Results

A total of 376 patients with a first episode of smear-positive pulmonary TB were registered in the health facilities. For 62 patients no informed consent could be obtained and 50 were excluded because they reported at least one risk factor for MDR-TB. Among the 264 eligible patients, 65 (24.6%) were not included because they did not leave a sample (n = 6), the primary culture was negative (n = 22), the subculture was negative (n = 36) or contaminated (n = 1). Thus, the total number of cultures from different subjects available for DNA genotyping was 199, representing 75.4% of eligible patients for this study. The male-to female ratio was 1.2; and median age was 31 years (range: 18 to 78).

### Drug-susceptibility test

Drug-Susceptibility Test (DST) results were available for 197 (99%) isolates. In total, 62 (31.5%) were resistant to at least one drug and 135 (68.5%) were fully susceptible (Table 1). Fifteen cases (7.5%) were MDR, 17 (8.6%) were monoresistant to H, 17 (8.6%) to S, and 1 (0.5%) was resistant to E (Table 1).

**Table 1:** **Resistance patterns of 197** ***M. tuberculosis *complex isolates**

**Type of resistance**	**n (%)**
Monoresistant	
H	17 (8.6)
R	0 (0.0)
S	17 (8.6)
E	1 (0.5)
Polyresistant (non-MDR)	
H + S	8 (4.1)
H + E	1 (0.5)
H + S + E	3 (1.5)
MDR	
H + R	5 (2.5)
H + R + S	8 (4.1)
H + R + E	1 (0.5)
H + R + S + E	1 (0.5)
Total resistant	62 (31.5)
Susceptible to all drugs	135 (68.5)
Total	197

*H* isoniazid, *R* rifampicin, *S* streptomycin, *E* ethambutol.

*MDR* multidrug-resistant, *n* number.

### Distribution of different lineages

**Figure 1:**
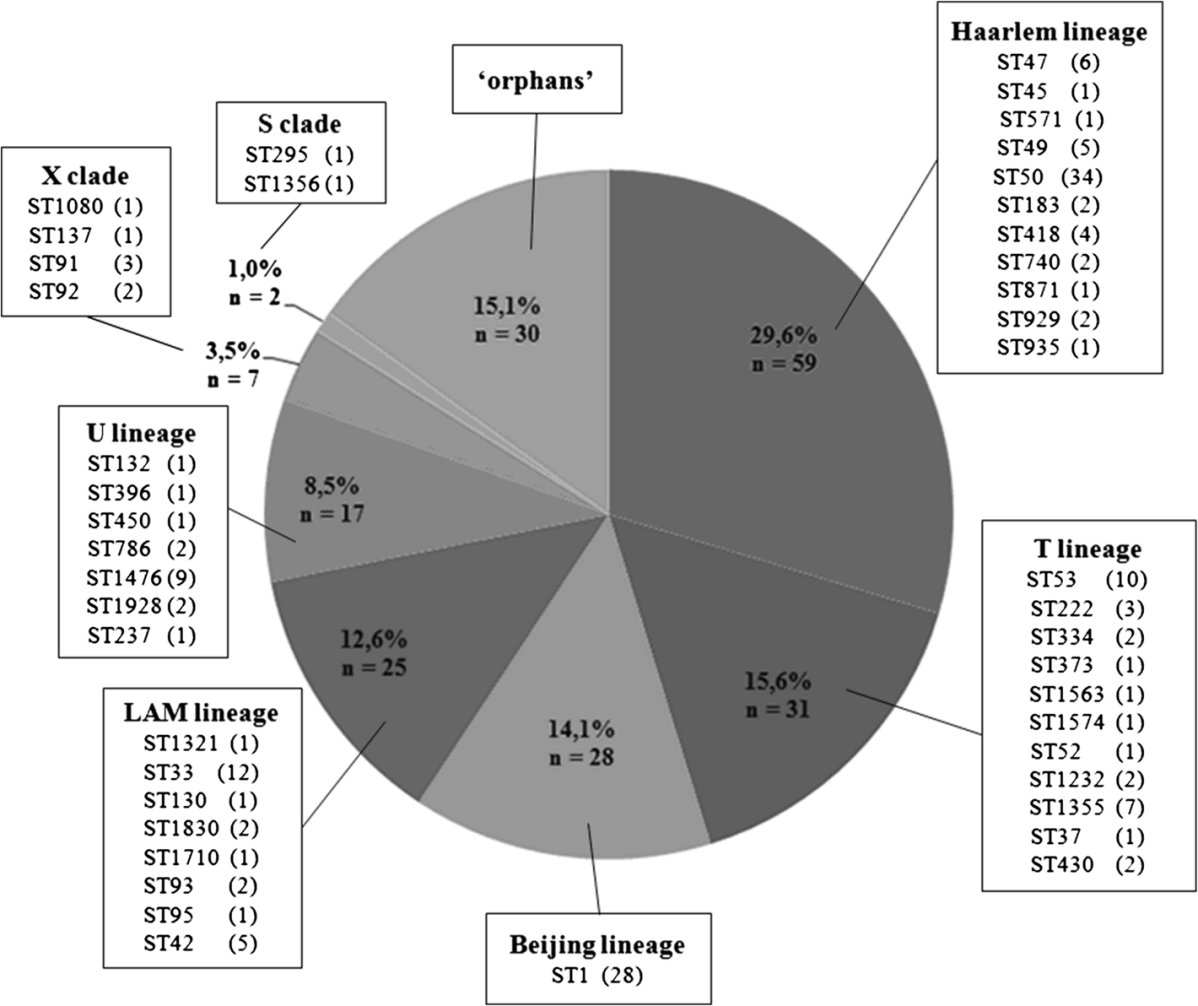
**Lineage and spoligotypes prevalence among the 199 isolates of *Mycobacterium tuberculosis *(n = number of isolates).**

Among the 199 typed isolates, patterns from 169 (84.9%) isolates belonged to seven lineages in the SpolDB4.0, whereas 30 (15.1%) isolates could not be matched to any lineage, and are thus referred to as “orphan”. Of these, 59 (34.9%) isolates belonged to the Haarlem lineage, while 110 (65.1%) were non Haarlem strains. Strains classified into non Haarlem lineage included strains from T lineage (15.6%), Beijing lineage (14.1%), Latin American Mediterranean (12.6%) and U lineage (8.5%). Few isolates (4.5%) belonged to the X and S clades (Figure 1).

### Predominant spoligotypes

A total of 44 different spoligotypes (ST) were obtained among the 199 isolates analyzed (Figure 1). Among these, the ST50 member of the Haarlem lineage was the most common (29.5%), followed by ST1 from the Beijing lineage (16.5%), ST33 from the Latin American Mediterranean lineage (7.1%) and ST53 from the T lineage (5.9%). Despite the relatively high HGDI of 91.8% [21], spoligotyping showed a clearly lower resolution compared to MIRU-VNTR typing.

No correlation was found between the identified lineages with regard to primary drug resistance when comparing MDR-TB versus non-MDRTB, non-MDR H-resistant versus H-susceptible isolates, and any H-resistant versus H-susceptible isolates. (see Table 2 for the most prevalent lineage). Similarly, none of the lineages showed an association with a specific age group, even though the Haarlem and Beijing lineages were slightly more prevalent among younger patients (p-value of 0.09 for Beijing and 0.07 for Haarlem, Table 2).

**Table 2:** Distribution of Haarlem and Non-Haarlem strains according to resistance and age group

	**Total**	**Haarlem**		**Non**		**p-value**
			**%**	**Haarlem**	**%**	
	**n = 199**	**n = 59**		**n = 140**		
**Resistance**						
profile *						
MDR	15	4	26.7	11	73.3	1.00
Non-MDR	182	54	29.9	128	70.1	
Non-MDR H-resistant	29	10	34.5	19	65.5	0.70
H-susceptible	168	48	28.6	120	71.4	
Any H-resistant	44	14	31.8	30	68.2	0.70
H-susceptible	153	44	28.8	109	71.2	
**Age groups**						
≤ 30 years	124	31	25.0	93	75.0	0.07
> 30 years	75	28	37.3	47	62.7	

*DST results were available for 197 isolates.

### MIRU-VNTR analysis

Unlike the spoligotype analysis, all isolates had a unique MIRU-VNTR pattern and we did not observe any case of bacterial subpopulation or mixed *M. tuberculosis* infection among our isolates.

## Discussion

Our study gives insights into the *M. tuberculosis* strains circulating among patients with no known risk factors for MDR-TB living in an area of high exposure to TB. We found that the Haarlem lineage was present in one third of the samples which is different from other Latin-American countries where the LAM lineage is the most predominant.

There are currently six phylogeographic lineages that make up the *M. tuberculosis* global population [22]. One is the Euro-American lineage, which includes all the spoligotypes predominating in the Western world (Haarlem, LAM, and the ill-defined T group) [11]. In particular, the Haarlem lineage is ubiquitous [23] and represents about 25% of the isolates in Europe, Central America, and the Caribbean, suggesting a link with the post-Columbus European colonization [24]. Haarlem strains have been responsible for a prolonged outbreak of multidrug-resistant (MDR) tuberculosis in Argentina [23,25] and are actively transmitted in urban settings in Colombia, causing major public health problems [26].

Contrary to previous reports [11,22,27-30] where the LAM lineage was the most common in Latin America, our results demonstrated that the Haarlem lineage, and to a lesser extent the T lineage, were the main circulating TB genotypes in patients with unknown risk factors. The Haarlem lineage was described in the Netherlands in 1999 [31]. Three main spoligotype-signatures define the variants H1, H2 and H3 [32]. In our study most of the Haarlem strains belonged to the H3-sublineage (84.7%) and just a few belonged to the H1-sublineage (13.6%). The Haarlem lineage is highly prevalent in Northern Europe and also in Central Africa, where it is believed to have been introduced during the European colonization, but it is present in the Caribbean to a lesser extent [33].

There are very few studies concerning the current genetic epidemiology of MTB strains circulating in Peru. Cohen *et al*. [34] focused on households in Lima in which >1 MDR-TB patient received treatment between 1996 and 2004; among the 391 MDR-TB isolates from 236 persons, the most prevalent lineages were LAM (n = 40.6%), T (n = 33.7%), Haarlem (n = 14.0%) and Beijing (n = 6.0%). Later on, Taype *et al*. [35] analyzed 323 sputum smear-positive samples collected between 2004 - 2006 from 3 districts in the north of Lima (Rímac, San Martin de Porres and Los Olivos districts) and found that the most prevalent lineages were LAM (23.8%), Haarlem (23.8%), and T (22.3%). Although the focus of these studies was different from ours (MDR-TB in one study and different districts in the other), and the sample size in each district studied remained small, these data suggest a trend for an increasing prevalence of the Haarlem genotype in the North-Eastern area of Lima. Further studies should focus on the emergence of the Haarlem lineage and its possible reasons.

As spoligotyping has limited resolution we typed the samples by 15-locus MIRU-VNTR. Interestingly, despite that strains were obtained from patients living in an area of high transmission, there was no evidence of a predominant clone. This could be due to selection of patients with low MDR-TB risk factors and the relatively short time window of sampling (7 months) for analyzing recent transmission [36,37].

When we stratified the genotypes by age, we observed that 62.3% corresponds to the youngest and most productive age group (≤30 years). Our results suggest that the Beijing and Haarlem lineages are slightly more frequent among younger people which might indicate their recent emergence in our study population. For Beijing this is in agreement with previous studies [38]. The observed differences were not statistically significant, but we cannot exclude that they might have been significant if the sample size would have been larger.

The predominance of the Haarlem genotype in our population assumes particular significance in light of previous studies demonstrating the ability of this genotype to cause outbreaks of MDR-TB, as reported in Argentina [23], the Czech Republic [39], Tunisia [40] and Poland [41]. Compared to the country-wide Peruvian data, a high MDR rate was found among new smear-positive pulmonary TB patient with unknown risk factors for MDR living in North Lima (5.3% vs. 7.5%, respectively) [4]. We also saw a high H-monoresistance rate (8.6%) and overall high H-resistance rate (22.3%) among the isolates, yet none of the lineages showed a significant association with primary MDR or H resistance.

Our study has some limitations. First, since this was a passive surveillance study, only patients attending health care facilities at the public sector were included. Second, the selection of patients with no known risk factors relied on patient’s self-reporting, which could have masked some risk factors, hence overestimating the MDR-TB rates among low risk patients. Third, the absence of clusters could be due to the relatively short study period and the exclusion of patients with risk factors.

## Conclusions

In summary, the population structure of *M. tuberculosis* complex among patients with no known risk factors for MDR-TB was diverse including 44 different spoligotypes but none of them was related to age or to any resistance pattern. Future prospective community-based studies should aim to accurately estimate the genetic variability of *M. tuberculosis* in high burden areas, and analyze their relationship with MDR-TB.

## Competing interests

The authors declare that they have no competing interests.

## Authors’ contributions

Conceived and designed the experiments: FB, LO, CS, LR. Performed the experiments: FB, JC, BA, LR. Analyzed the data: FB, LO, JC, BA, BCJ, LR. Wrote the paper: FB, LO, BCJ, CS, LR. All authors read and approved the final manuscript.

## Pre-publication history

The pre-publication history for this paper can be accessed here:

http://www.biomedcentral.com/1471-2334/13/397/prepub
